# Peroxidase-like MoS_2_/Ag nanosheets with synergistically enhanced NIR-responsive antibacterial activities

**DOI:** 10.3389/fchem.2023.1148354

**Published:** 2023-03-09

**Authors:** Huiying Chen, Xinshuo Zhao, Bingbing Cui, Haohao Cui, Mengyang Zhao, Jun Shi, Jingguo Li, Zhan Zhou

**Affiliations:** ^1^ Henan Provincial People’s Hospital, People’s Hospital of Zhengzhou University, Zhengzhou, China; ^2^ School of Materials Science and Engineering, Zhengzhou University, Zhengzhou, China; ^3^ Henan Key Laboratory of Function-Oriented Porous Materials, College of Chemistry and Chemical Engineering, Luoyang Normal University, Luoyang, China

**Keywords:** two-dimensional, silver nanoparticles, photo-responsive, peroxidase-like activity, synergistic antibacterial

## Abstract

Pathogenic microbial infections have been threatening public health all over the world, which makes it highly desirable to develop an antibiotics-free material for bacterial infection. In this paper, molybdenum disulfide (MoS_2_) nanosheets loaded with silver nanoparticles (Ag NPs) were constructed to inactive bacteria rapidly and efficiently in a short period under a near infrared (NIR) laser (660 nm) in the presence of H_2_O_2_. The designed material presented favorable features of peroxidase-like ability and photodynamic property, which endowed it with fascinating antimicrobial capacity. Compared with free MoS_2_ nanosheets, the MoS_2_/Ag nanosheets (denoted as MoS_2_/Ag NSs) exhibited better antibacterial performance against *Staphylococcus aureus* by the generated reactive oxygen species (ROS) from both peroxidase-like catalysis and photodynamic, and the antibacterial efficiency of MoS_2_/Ag NSs could be further improved by increasing the amount of Ag. Results from cell culture tests proved that MoS_2_/Ag3 nanosheets had a negligible impact on cell growth. This work provided new insight into a promising method for eliminating bacteria without using antibiotics, and could serve as a candidate strategy for efficient disinfection to treat other bacterial infections.

## 1 Introduction

Bacterial infection, a major case resulting in pathological disorder, remains challenging to treat in terms of its highly horrible morbidity and mortality (Fisher et al., 2017; Qi et al., 2017). With the discovery and application of penicillin, human health and care present prosperous prospects in the field of fighting against microbes. However, bacteria continue to evolve and the abuse of anti-biotic leads to rapid and widespread drug resistance (Roope et al., 2019; Li et al., 2023a), which results in the increasingly difficult treatment of bacterial infections. Antibiotics administration against the bacterial infections can no longer catch up with the pace of bacterial evolution (Chen et al., 2023). According to statistics, 600 to −700 species of microbes have been identified on the planet, and with the bacteria still evolving and mutating, bacterial infections remain one of the greatest challenges to human health (Bakkeren et al., 2020; Li et al., 2021). It is imperative to develop a novel and favorable antimicrobial strategy with desired properties such as antibiotic-free, and biocompatible.

Due to the minimal likelihood of developing drug resistance and indeed the reliance on photo-responsive materials to carry out the operation (Zhu et al., 2020; He et al., 2022b), the photodynamic antibacterial method has drawn tremendous attention in microbiological applications (Yang et al., 2018; Chauhan et al., 2019; Chen et al., 2019; Han et al., 2020; Zhao et al., 2022b). Numerous cutting-edge substances, including nano-metal oxides (Ning et al., 2017; Jin et al., 2019; He et al., 2022a), two-dimensional (2D) materials (Liu et al., 2016; Zhao et al., 2023), and other photosensitive substances (Liu et al., 2019), can produce electron-hole pairs when exposed to certain wavelengths of light. The ejected electrons can then be captured by the oxygen in the environment to create reactive oxygen species (ROS), which can cause the death of pathogenic or diseased cells (Chen et al., 2022a; Zhou et al., 2022b; Hu et al., 2023). In particular, versus traditional chemotherapy, photodynamic therapy (PDT) manages to avoid the progression of drug resistance by primarily acting its biocidal activity through the oxidative damage of biological macromolecules such as lecithin, enzymes, nutrients, and DNA in cell membranes (Zhang et al., 2022b; Xiu et al., 2022).

MoS_2_ has been utilized in the departments of catalysis (Wu et al., 2022), drug delivery (Zhang et al., 2019a), and biomedicine (Yadav et al., 2019; Hu et al., 2022b) for the sake of its superior biocompatibility, high specific surface area, ultrathin atomic layer structure in two dimensions (2D) (Huang et al., 2022). Additionally, the photo-responsive properties of MoS_2_ from UV to near-infrared light, and the human-friendly elements of sulfur and molybdenum make it available for photodynamic therapy (Zhou et al., 2022a; Sethulekshmi et al., 2022). However, the small band gap of MoS_2_ makes it easier for electron-hole pairs to combine, which can reduce its photodynamic activity (Zhu et al., 2020). Hence, it is required to identify other appropriate materials to combine with MoS_2_. By accelerating the movement of electrons, the combination of precious metals with semiconductor materials can considerably enhance the photodynamic characteristics of semiconductor materials (Xia et al., 2015; Raza et al., 2017). The superior conductivity and inherent antibacterial properties of Ag make it a suitable precious metal material (Li et al., 2022b). More importantly, the Ag also have the effect of surface plasmon resonance, which allows electrons to escape from the outermost surface and be captured by the surrounding oxygen to produce ROS (Zhu et al., 2020). The local ^1^O_2_ produced by MoS_2_ with 660 nm laser irradiation can create a highly oxidized environment for the activation of metal nanoparticles, resulting in highly toxic metal ions that can induce oxidative stress to kill bacteria (Wei et al., 2021; Hu et al., 2022a; Li et al., 2022a; Xue et al., 2023). Another advantage of MoS_2_ is that it can produce ROS when reacts with hydrogen peroxide (H_2_O_2_) (Li et al., 2023b), hastening the death of bacteria (Zhao et al., 2015). Therefore, combining the characteristics of photodynamic and peroxidase, nanocomposites based on MoS_2_ nanosheets could provide a promising alternative for resistant bacterial infections.

**SCHEME 1:**
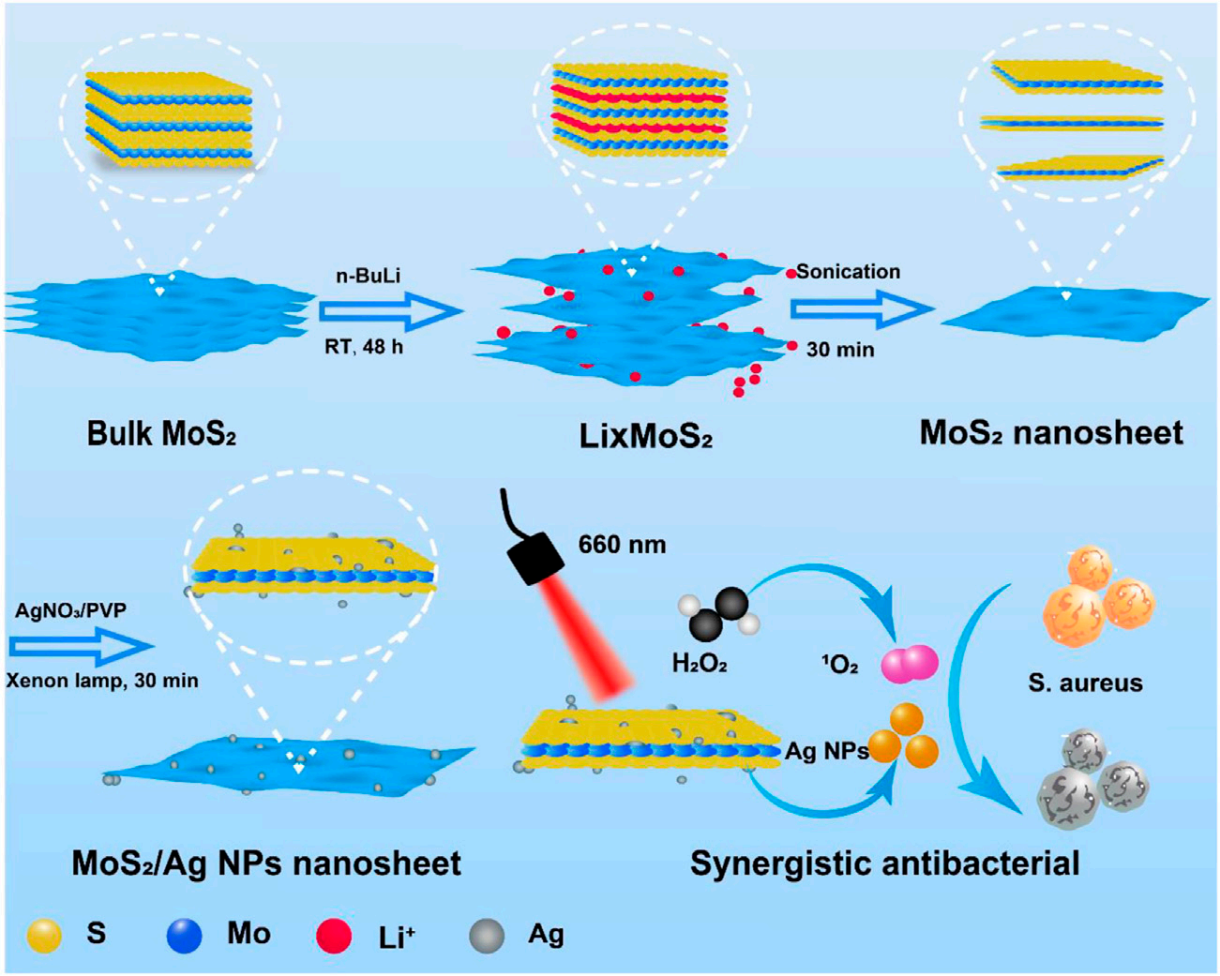
The schematic diagram for the preparation and synergistic antibacterial of MoS_2_/Ag nanosheets.

Herein, MoS_2_/Ag nanosheets (denoted as MoS_2_/Ag NSs) with rapidly adjustable antibacterial properties was constructed by a simple chemical exfoliation and xenon lamp reduction method (Scheme 1). Ag NPs and MoS_2_ together increased the energy evolution routes and electron transport at the interface, considerably raising the photodynamic activity of MoS_2_ and, as a result, the amount of ROS produced when irradiated by 660 nm near-infrared light. Meanwhile, a low concentration of H_2_O_2_ could be catalyzed into detrimental ROS by the intrinsic peroxidase-like property of MoS_2_/Ag NSs, which was vital in attacking the bacterial membranes. As a result, this work displayed remarkable promise in the practical treatment of inflammatory diseases while also offering a promising strategy for swift and efficient sterilization.

## 2 Experimental details

### 2.1 Chemicals

Molybdenum disulfide (MoS_2_) layered bulk crystals, n-Butyllithium (2.5 M in cyclohexane) were purchased from Sigma Aldrich (United States). Polyvinylpyrrolidone (PVP) with the average MW 58000 was purchased from Aladdin Reagent Company (China). AgNO_3_ was obtained from Macklin. Ag nanoparticles were successfully grown on MoS_2_ nanosheets by 300 W xenon lamp (PLS-SXE100) irradiation. 3-(4,5-Dimethylthiazol-2-yl)-2,5-diphenyl tetrazolium bromide (MTT) was provided from Sigma Biochemical Technology Co., Ltd. (Shanghai, China). LIVE/DEAD™ Baclight™ Bacterial Viability Kit (L7012) was purchased from Invitrogen (China). Cell counting Kit-8 (CCK-8) was purchased from Beyotime (China). *Staphylococcus aureus* and L929 cells were supplied by the Henan Eye Institute.

### 2.2 Characterization

Scanning electron Microscope (SEM) images were captured Field Launch Scanning Electron Microscope (Zeiss Sigma 500, Germany). Transmission electron microscope (TEM) images taken on by field emission transmission electron microscope (JEM-2100F, Japan). The diameter of particles and zeta potential of MoS_2_/Ag NSs were detected by Malvern ZEM 3700 equipment. The antibacterial property of samples with MTT was investigated by 2,104 Multilabel Microplate Reader (PerkinElmer). UV-Vis-NIR spectra were detected with a spectrophotometer from Agilent Technologies (Cary 5,000). Fluorescence pictures of microbial were observed with a Nikon 80i fluorescence microscope.

### 2.3 Synthesis of MoS_2_ nanosheets

The similar approach outlined in the prior research was used to prepare the MoS_2_ nanosheets (Chen et al., 2022b). The specific steps were as follows: the grinded MoS_2_ crystals (100 mg) were immersed in a solution of n-butyllithium (2.5 M in cyclohexane, 5 mL) and kept in glove boxes for 48 h to obtain lithium intercalation compounds. The precipitate at the bottom was washed three times with hexane after taking of the upper layer of n-butyllithium, followed by adding 50 mL of water and sonicated for 30 min to produce a homogeneous suspension. The large-size nanosheets were removed by centrifuging at 5,000 rpm for 10 min. The supernatant was further centrifugation products at 5,000–12000 rpm were collected and washed three times with DI water to obtain MoS_2_ nanosheets.

### 2.4 Preparation of MoS_2_/Ag NSs

40 mg PVP was added to 20 mL of synthesized MoS_2_ suspension in triplicate, and the AgNO_3_ (1 mg/mL) solution with various volumes (0.5, 1, and 2 mL) was added to the mixture. After stirring evenly, the mixture was lit under a xenon lamp at 300 W for 30 min. The crude products with different silver content were denoted as MoS_2_/Ag1, MoS_2_/Ag2, and MoS_2_/Ag3 and were washed two times with DI water to get the MoS_2_/Ag nanosheets with different silver contents.

### 2.5 Cytotoxicity tests

Using the Cell Counting Kit-8, the cytotoxicity of MoS_2_/Ag3 NSs was determined (CCK-8, Beyotime). Each well plate was injected with 7 to 8 10^3^ L929 cells for 24 h in a thermostatic incubator set to 37°C with 5% CO_2_, and then the wells were filled with 100 μL of the MoS_2_/Ag3 NSs solution for another 24 h. Subsequently, a fresh medium (100 μL) containing 10% CCK-8 agentia was added. This medium was then cultured for 4 h in a thermostatic incubator. Enzyme labeling (PerkinElmer Envision, England) was used to detect the optical density and cell viability at 450 nm.

### 2.6 Antibacterial test

*Staphylococcus aureus* (*S. aureus*) was used as the bacterial model. Frozen strains were first resuspended and transferred to columbia blood agar plates to be incubated overnight in a 37°C incubator, and then took a single colony by inoculation loops and transferred it to 4 mL LB medium and incubated for 12 h at 220 rpm on a shaker. Bacterial suspension was diluted to 10^7^ CFU/mL for inhibition experiments. Microdilution, plate counting, fluorescence staining, and scanning electron microscopy were used to examine the antibacterial activity and mechanism of MoS_2_/Ag nanosheets against *S. aureus*.

#### 2.6.1 Plate colony counting assay

The plate colony counting assay was employed to investigate the antibacterial performance. Firstly, *S. aureus* was mixed with different concentrations (10, 20, 30, 40, 50 μg/mL) of sample groups MoS_2_, MoS_2_/Ag1, MoS_2_/Ag2, MoS_2_/Ag3 in 96-well plates, respectively. All groups were irradiated with a 660 nm laser for 10 min and treated with hydrogen peroxide (H_2_O_2_) at a final concentration of 100 μM. Furthermore, *S. aureus* treated with H_2_O_2_ under laser irradiation was taken as the control. To further determine the effect of H_2_O_2_ or laser on the antimicrobial properties of the materials, four groups of each kind of material: I) bacteria control, II) bacteria + H_2_O_2_, III) bacteria + material, IV) bacteria + material + H_2_O_2_ were treated without or with NIR laser (660 nm) irradiation for 10 min. All experiments were carried out according to the following procedure, after culturing at 37°C for 3 h, 100 µL of diluted bacterial suspension was taken out and evenly spread on the blood agar plates and placed at 37°C for 12 h. The antibacterial ability of the material was determined by the colonies that grew on the blood agar plates. Each experiment was conducted at least three times.

#### 2.6.2 The cell viability of *S. aureus* assay

The premise of the test is that tetrazole is converted to blue crystalline methionine by the enzyme succinate dehydrogenase of live cells (Cheng et al., 2022). The percentage of living cells is proportional to the amount of blue-purple crystalline methionine produced. By using a microplate equipment to evaluate the optical density (OD_600_) of various holes, the bacterial survival rate was examined by comparing the optical density data.

Briefly, MTT solution (10 μL, 5 mg/mL) was mixed with *S. aureus* treated with the material and hatched in a constant temperature incubator at 37°C for 4 h. The survival rate of *S. aureus* was obtained by detecting the value of optical-density at 600 nm for 0 and 4 h with a micropore meter.

#### 2.6.3 Live/dead *S. aureus* staining experiment

SYTO™-9 is a green fluorescent nucleic acid dye that can penetrate cell membranes. PI is a nuclear stain that can penetrate the damaged cells, making the nucleus red. PBS or MoS_2_/Ag3 nanosheets (30 μg/mL) were mixed with a suspension of *S. aureus* treated by laser or H_2_O_2_ (100 μM), respectively. After being exposed to a 660 nm laser for 10 min, the cells were placed in an incubator at a constant temperature for 3 h, and the combination was then incubated at ambient temperature for 15 min without illumination. The stained *S. aureus* suspension (10 μL) was put on a slide, covered with the coverslip, and the excess dye was removed. Finally, bacterial staining images were observed by using a fluorescence microscope.

#### 2.6.4 Micromorphology of bacteria

*S. aureus* under different conditions were collected by centrifugation (4,000 rpm, 5 min). The *S. aureus* was fixed with 2.5% glutaraldehyde for 12 h and washed twice with PBS at room temperature. Next, the bacteria were treated with 10%, 25%, 50%, 75%, and 100% ethanol successively and gradually dehydrated for 15 min. For SEM observation, the final bacterial solution was dropped onto a silica substrate and sprayed with gold.

## 3 Results and discussion

### 3.1 Preparation and characterization of MoS_2_/Ag NSs

**FIGURE 1:**
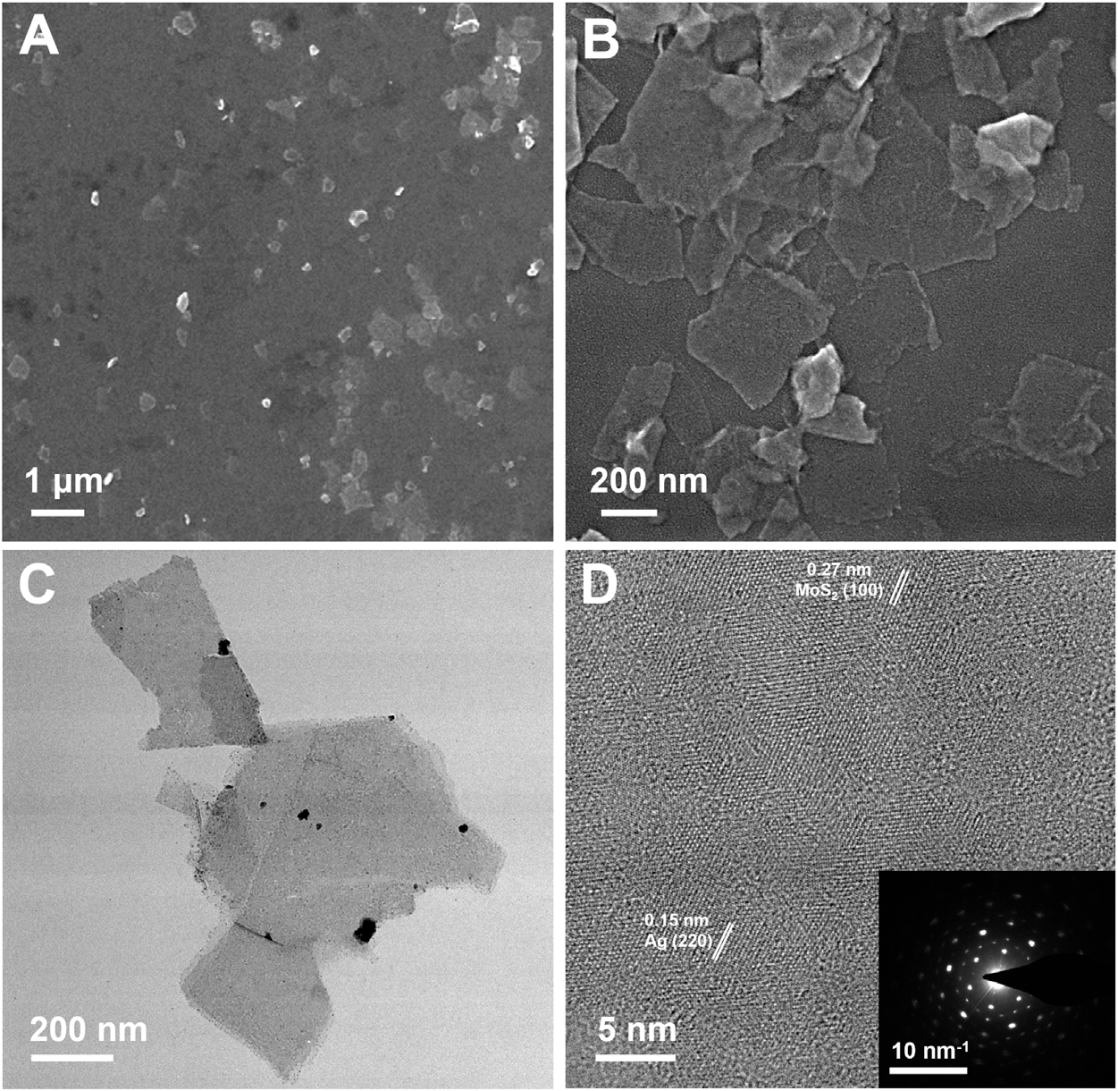
**(A, B)** SEM, **(C)** TEM, and **(D)** HR-TEM images of MoS_2_/Ag3 nanosheets. Inset in **(D)** is the SAED pattern of MoS_2_/Ag3 nanosheets.

The synthesis process of MoS_2_/Ag nanosheets was schematically stated in Scheme 1. The MoS_2_ nanosheets were fabricated and obtained according the previous procedure (Chen et al., 2022b). To load the silver nanoparticles (Ag NPs) onto the surface of MoS_2_ nanosheets, AgNO_3_ was first mixed with MoS_2_ nanosheets solution and then reduced under the irradiation of a Xenon lamp, the highly dispersed Ag NPs could be obtained. The scanning electron microscope (SEM) images clearly showed that the MoS_2_/Ag3 NSs exhibited the uniform nanosheet morphology with a size around 200–400 nm **(** Figures 1A, B **)**. The TEM images disclosed that the Ag NPs were successfully dispersed on the surface of MoS_2_ nanosheets (Figure 1C), and its high-resolution transmission electron microscopy (HRTEM) image (Figure 1D) further presented that the lattice fringes of 0.27 and 0.15 nm belonged to planes of MoS_2_ (100) and Ag (220), respectively, indicating Ag NPs were successfully loaded onto the surface of nanosheets. Promisingly, the selected area electron diffraction (SAED) pattern of MoS_2_/Ag3 NSs was characterized by the presence of bright diffraction spots with regular hexagon (inset of Figure 1D), signifying its single-crystalline nature.

**FIGURE 2:**
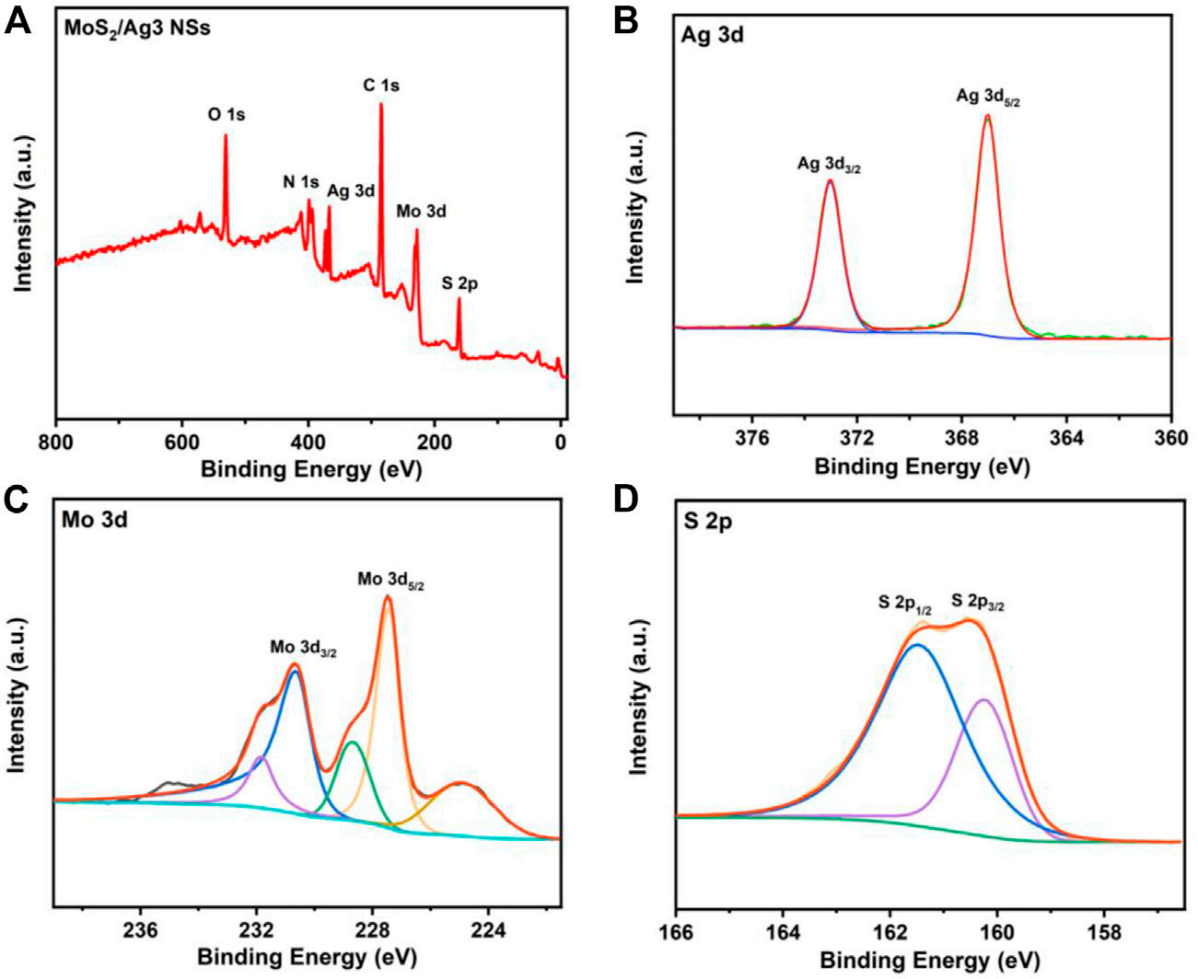
**(A)** X-ray photoelectron spectra of MoS_2_/Ag3 NSs. **(B)** Ag 3d, **(C)** Mo 3d, and **(D)** S 2p spectra of MoS_2_/Ag3 NSs.

Both the MoS_2_ and MoS_2_/Ag3 NSs were further characterized by X-ray photoelectron spectroscopy (XPS). The XPS survey spectra revealed the presence of six elements (C, N, O, S, Mo, and Ag) in MoS_2_/Ag3 NSs (Figure 2A), while the MoS_2_ nanosheets were found to lack the Ag element (Supplementary Figure S1A). Compared with the XPS Ag 3d spectrum of MoS_2_ nanosheets (Supplementary Figure S1B), that of MoS_2_/Ag3 NSs exhibited two distinct peaks at 372.98 eV and 366.98 eV corresponding to Ag 3d_3/2_ and Ag 3d_5/2_ of Ag (0) (Figure 2B) (Qiao et al., 2017; Li et al., 2022a), which indicated the fabrication of MoS_2_/Ag NSs. As shown in Figure 2C, the high-resolution XPS Mo 3d spectrum of the MoS_2_/Ag3 NSs given two main peaks at 230.68 eV and 227.48 eV, which belonged to Mo 3d_3/2_ and Mo 3d_5/2_ of Mo (IV), respectively (Chen et al., 2022a). The characteristic peaks at 161.38 eV and 160.48 eV (Figure 2D) originated from S 2p_1/2_ and S 2p_3/2_ of S (II) (Zhou et al., 2020), respectively. The UV-Vis absorption spectroscopy data (Supplementary Figure S2) were used to confirm that Ag NPs had been synthesized without structural alteration of MoS_2_. Moreover, it was found that the augmentation of silver content had negligible effect on particle size and Zeta potential of MoS_2_/Ag3 NSs (Supplementary Figure S3).

### 3.2 Antibacterial activity *in vitro*

**FIGURE 3:**
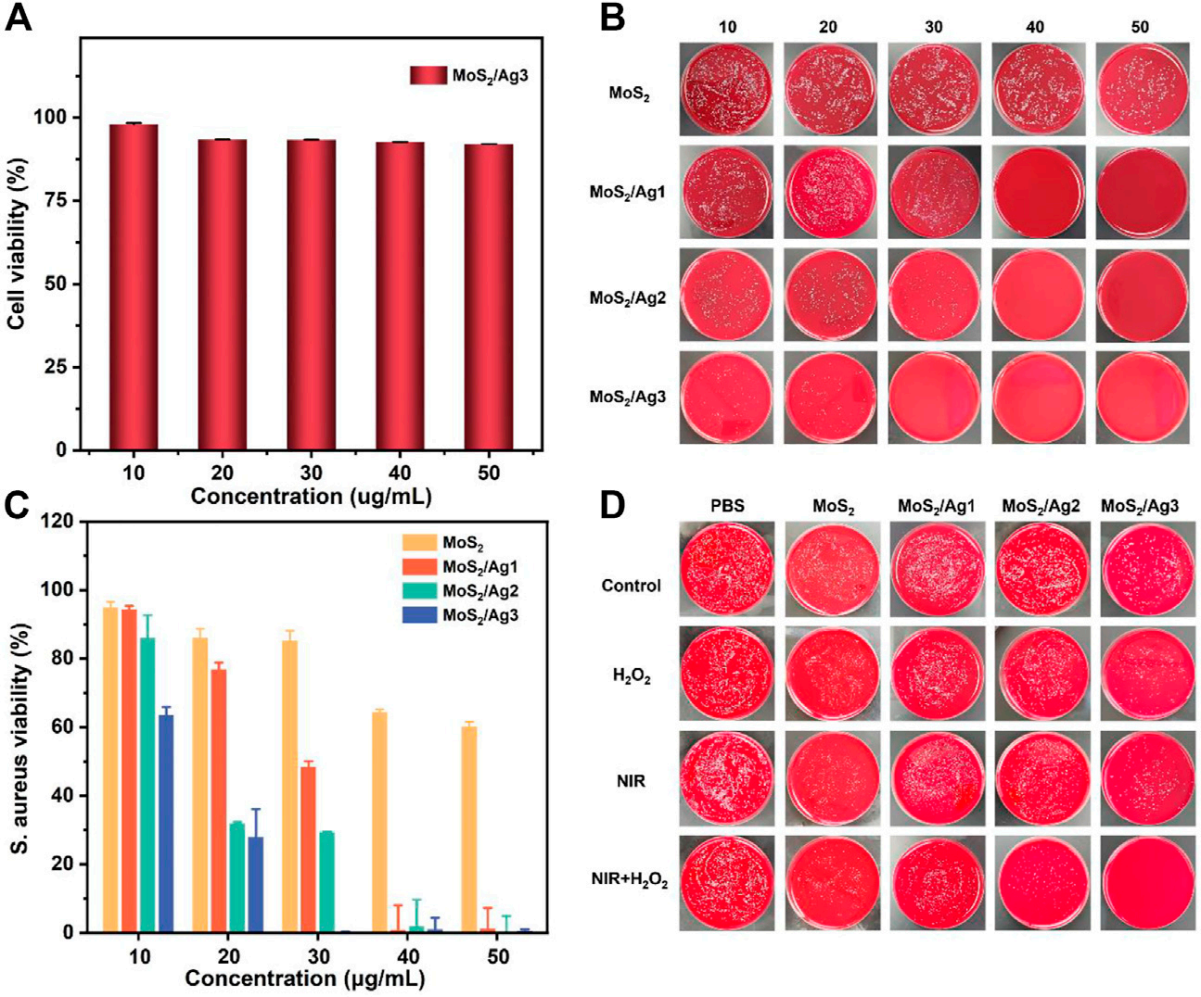
**(A)** Cell viability of L929 cells after treated with MoS_2_/Ag3. **(B)** *S. aureus* bacterial colony development after treating with various doses of MoS_2_, MoS_2_/Ag1, MoS_2_/Ag2, and MoS_2_/Ag3, respectively. **(C)** Bacterial survival rate of *S. aureus* after treatment with several groups. All groups were treated with H_2_O_2_ (100 µM) under the 660 nm irradiation for 10 min (1 W/cm^2^). **(D)** *S. aureus* bacterial colony development after treatment with different groups (PBS, MoS_2_, MoS_2_/Ag1, MoS_2_/Ag2, MoS_2_/Ag3) in the conditions of H_2_O_2_, NIR, NIR + H_2_O_2_, respectively.

As shown in Figure 3A, we evaluated the cytotoxicity of MoS_2_/Ag NSs with the highest silver content (MoS_2_/Ag3) by CCK-8 experiment in L929 cells. The viability of L929 cells was still higher than 85% after the treatment with MoS_2_/Ag3 at the concentration of 50 μg/mL, showing that excellent biocompatibility and the great potential for *in vivo* and *in vitro* antibacterial applications.

Considering its great photodynamic performance and promising peroxidase-like ability (Supplementary Figure S4), the germproof capacity against *S. aureus* was further appraised by plate counting method. After co-incubation with the designed materials, it was found that the group of H_2_O_2_ or laser irradiation had a negligible antimicrobial effect against *S. aureus* (Figure 3B). Therefore, the optimal inhibitory concentration of all samples was determined under laser irradiation at the presence of H_2_O_2_. As presented in Figure 3B, the visual colony dramatically decreased with increasing concentrations of antibacterial agents, which showed the fascinating concentration-dependent bactericidal capacity. It was noting that the antibacterial effect was further enhanced with increasing density of Ag NPs (30 μg/mL), implying that maybe more ROS were activated to grievously destroy the morphology of bacteria. The antibacterial properties of MoS_2_ NSs were also determined in terms of MTT assay. The results demonstrated that only a survival rate of 0.12% was performed in the group of MoS_2_/Ag3 with the concentration of 30 μg/mL, but obvious colonies were observed in other groups (Figures 3B, C). Interestingly, an impressive bactericidal effect against *S. aureus* was observed when the concentration of MoS_2_/Ag nanosheets was raised.

In order to intuitively observe the inhibitory effect against *S. aureus* under irradiation of laser (660 nm) or in the presence of H_2_O_2_, we kept up with the plate-counting antibacterial experiment with MoS_2_/Ag nanosheets at the concentration of 30 μg/mL. It has been reported that H_2_O_2_ (100 μM) together with near-infrared irradiation exhibited hardly inhibitory effect (Wei et al., 2021). Although the growth of microorganisms was not completely suppressed, H_2_O_2_ or laser irradiation had a more conspicuous antibacterial effect than only material in other groups (Figure 3D), suggesting that the ROS was insufficient to kill *S. aureus* at the current concentration. Following the combination of laser irradiation and H_2_O_2_ therapy, *S. aureus* survival rate continued to decline, indicating synergistically antibacterial capacity with regard to photodynamic therapy and peroxidase-like strategy (Li et al., 2020; Zhang et al., 2022a). Compared to the other groups, MoS_2_/Ag3 exhibited the most robust antimicrobial ability and almost all bacteria were inactivated, demonstrating the antibacterial effectiveness might be improved with the addition of Ag. Thus, it could be speculated that the practical bactericidal process under irradiation was dominated by the synergistic impact of MoS_2_ and Ag NPs to boost ROS formation.

**FIGURE 4:**
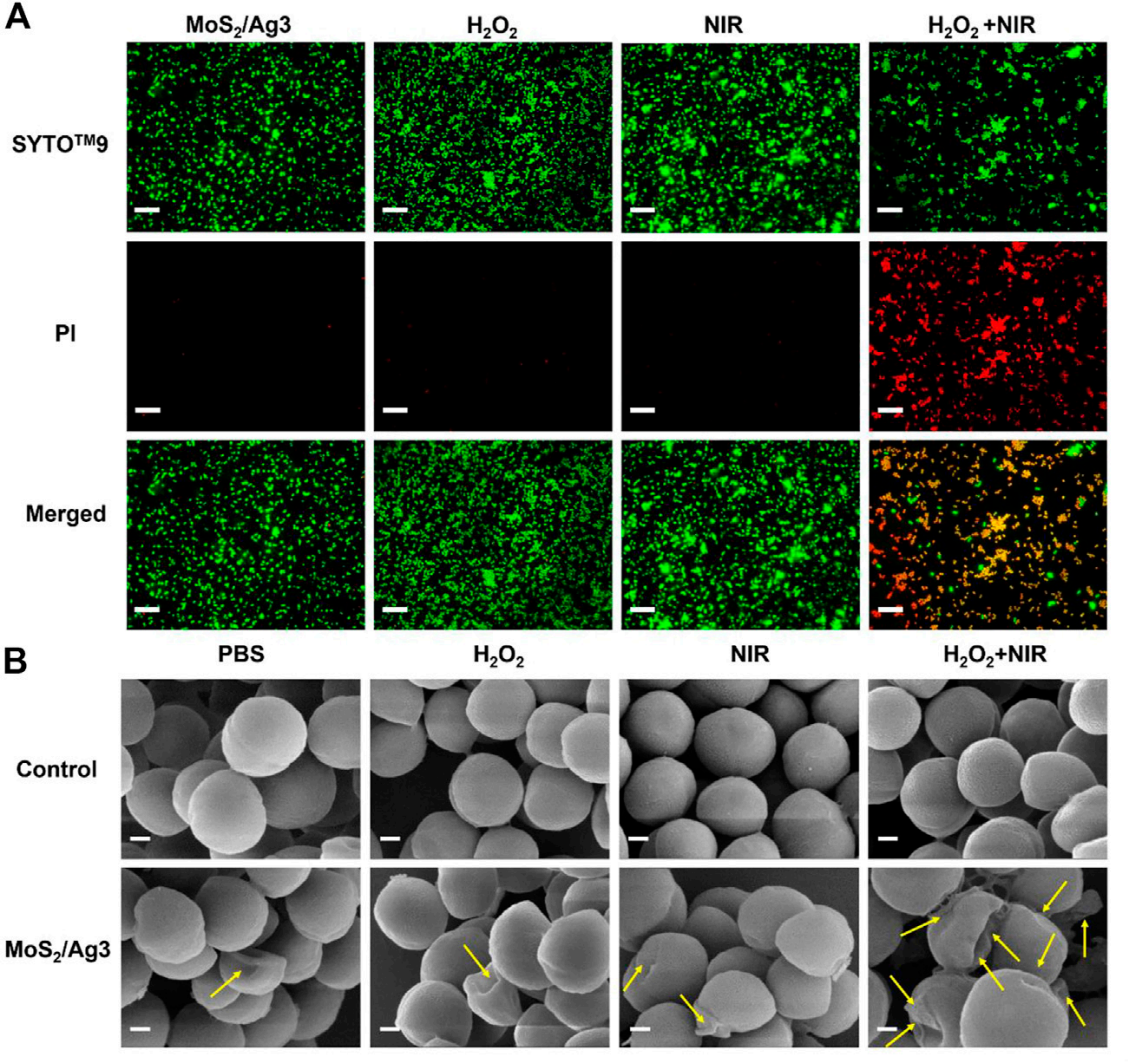
**(A)** Fluorescent images of *S. aureus* incubated with different conditions (MoS_2_/Ag3 (30 μg/mL), MoS_2_/Ag3 (30 μg/mL) + H_2_O_2_ (100 μM), MoS_2_/Ag3 (30 μg/mL) + H_2_O_2_ (100 μM) + NIR (irradiation by a 660 nm laser with 1 W/cm^2^ for 10 min), Scale bar: 5 μm. **(B)** SEM pictures of *S. aureus* after various treatments, Scale bar: 200 nm.

MoS_2_/Ag3 NSs (30 μg/mL) was utilized to carry out the live-dead bacteria staining experiment for the sake of investigating the antibacterial mechanism. All *S. aureus* could be stained by green fluorescence (SYTO™-9) and only damaged *S. aureus* presented red fluorescence (PI). Fluorescence images showed (Supplementary Figure S5) that membranes of *S. aureus* would not be destroyed in terms of free H_2_O_2_, NIR irradiation (10 min), or combination of H_2_O_2_ and NIR irradiation (10 min). Since the red fluorescence in the H_2_O_2_ condition only was practically identical to that of a single laser irradiation in Figure 4A, so it is almost harmless to conduct independent action. But the peroxidase-like activity of MoS_2_/Ag3 NSs could kill off large numbers of bacteria in the presence of H_2_O_2_, implying that the synergistic effect made *S. aureus* unable to survive.

To further appraise the antimicrobic capacity of MoS_2_/Ag3 NSs, surface morphologies of antibacterial with different treatment were detected by SEM. It was observed that the bacteria of the PBS group maintained a smooth and unbroken membrane, meanwhile the *S. aureus* treated by H_2_O_2_ or NIR revealed no evident difference with the PBS group, suggesting the intact structure of *S. aureus* (Figure 4B). However, obvious wrinkles and destruction of *S. aureus* were observed in the MoS_2_/Ag3, MoS_2_/Ag3 + H_2_O_2_, and MoS_2_/Ag3 + NIR groups (Figure 4B). Promisingly, the bacterial membranes shrunk more seriously and even destroyed (signaled by yellow arrows) in the MoS_2_/Ag3 + H_2_O_2_ + NIR group (Figure 4B), which attributed to the ample ROS synergistically generated by the catalysis of MoS_2_/Ag3 under the NIR laser irradiation in the presence of H_2_O_2_.

**FIGURE 5:**
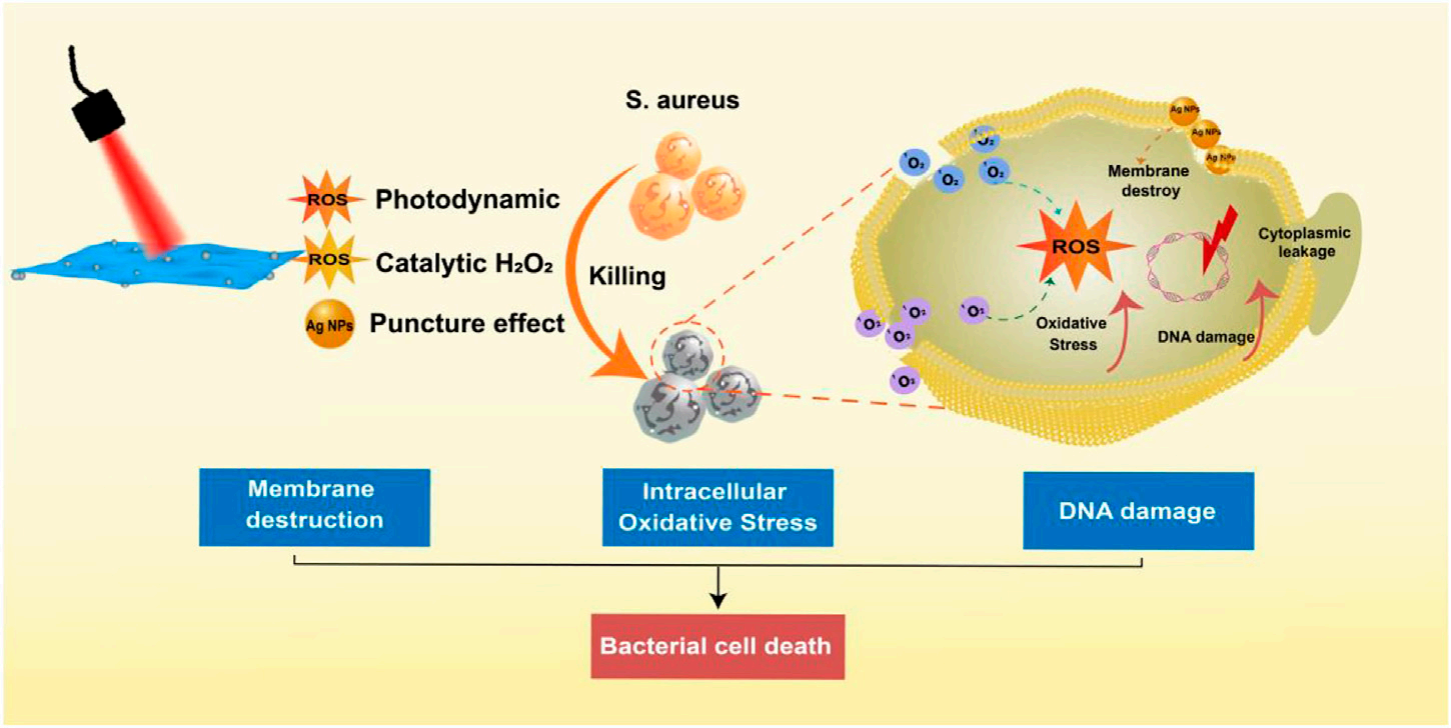
The multi-level synergistic antibacterial mechanism of MoS_2_/Ag nanosheets.

Inspired by the results of the *in vitro* antibacterial assays described above, we hypothesized a multi-level synergistic antibacterial mechanism **(** Figure 5 **)**. The sterilization mechanism of Ag NPs was mainly to puncture the cell membrane of their tiny size (Akter et al., 2018). When irradiated by a 660 nm laser, the valence band electrons of MoS_2_ were stimulated to change into the conduction band and produce electron-hole pairs (Zhu et al., 2020). Additionally, combining Ag NPs and MoS_2_ could facilitate electron transport and prevent the compounding of electron-hole pairs, generating a significant amount of photoelectrons and holes (Ma et al., 2016). Virtually, the positively charged holes had a robust oxidability and could yield ^1^O_2_ when reacted with oxygen (Karkhanechi et al., 2014; Zhang et al., 2020; Zhao et al., 2022a; Luo et al., 2022). Importantly, H_2_O_2_ could be catalyzed to produce ROS for the sake of the peroxidase-like ability of MoS_2_ (Wang et al., 2016). As a result, when bacteria were exposed to near-infrared laser, the synergistic action assaulted the bacterial membrane, causing the bacterial metabolic barrier and ultimately causing bacterial mortality.

## 4 Conclusion

In conclusion, a facile artificial nanosheet of MoS_2_/Ag was designed by a simple method for synergistic photodynamic and peroxidase-like catalytic antibacterial treatment. The developed MoS_2_/Ag nanosheets may effectively inactivate bacteria by producing poisonous ROS supported to assault the membranes. Importantly, MoS_2_/Ag3 nanosheets exhibited an antimicrobial efficiency of 99.88% against *S. aureus* within 10 min under 660 nm illumination in the presence of H_2_O_2_. Owing to the modification of PVP, the biocompatibility of MoS_2_/Ag3 NSs was significantly improved. This particular type of photo-responsive material demonstrated prominent effectivity in antimicrobial treatment with minimal cytotoxicity, which could serve as a promising candidate of antibiotic-free treatment of bacterial infections.

## Data Availability

The original contributions presented in the study are included in the article/Supplementary Material, further inquiries can be directed to the corresponding authors.
