# Supplementary material for: Peroxidase-like MoS2/Ag nanosheets with synergistically enhanced NIR-responsive antibacterial activities
Source: Front Chem. 2023 Mar 9;11:1148354. doi: 10.3389/fchem.2023.1148354 (PMC10033522; doi:10.3389/fchem.2023.1148354)
Supplement: Supplementary file 1 [file DataSheet1.DOC]

**Peroxidase-like MoS_2_/Ag nanosheets with synergistically enhanced NIR-responsive antibacterial activities**

Huiying Chen,^1,3^ Xinshuo Zhao,^2^ Bingbing Cui^1,3^, Haohao Cui,^1,3^ Mengyang Zhao^1^, Jun Shi^3^, Zhan Zhou^1,2*^ and Jingguo Li^1*^

^1^Henan Provincial People’s Hospital, People’s Hospital of Zhengzhou University, Zhengzhou 450003, PR China

^2^College of Chemistry and Chemical Engineering, Henan Key Laboratory of Function-Oriented Porous Materials, Luoyang Normal University, Luoyang, 471934, PR China

^3^School of Materials Science and Engineering, Zhengzhou University, Zhengzhou 450001, People’s Republic of China

*Corresponding author. Email: [zhouzhan@lynu.edu.cn](mailto:zhouzhan@lynu.edu.cn); [lijingguo@zzu.edu.cn](mailto:lijingguo@zzu.edu.cn)


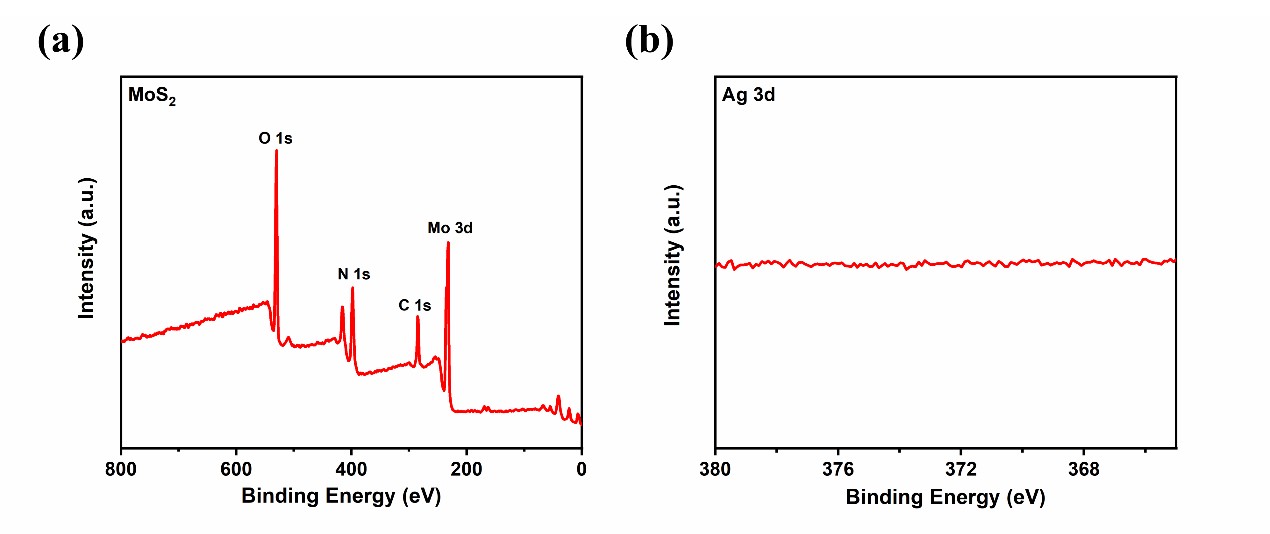
**Fig. S1**. (a) X-ray photoelectron spectra of MoS_2_ nanosheets. (b) Ag 3d spectra of MoS_2_





**Fig. S2.** The UV–vis light absorption spectra of MoS_2_, MoS_2_/Ag1, MoS_2_/Ag2, MoS_2_/Ag3


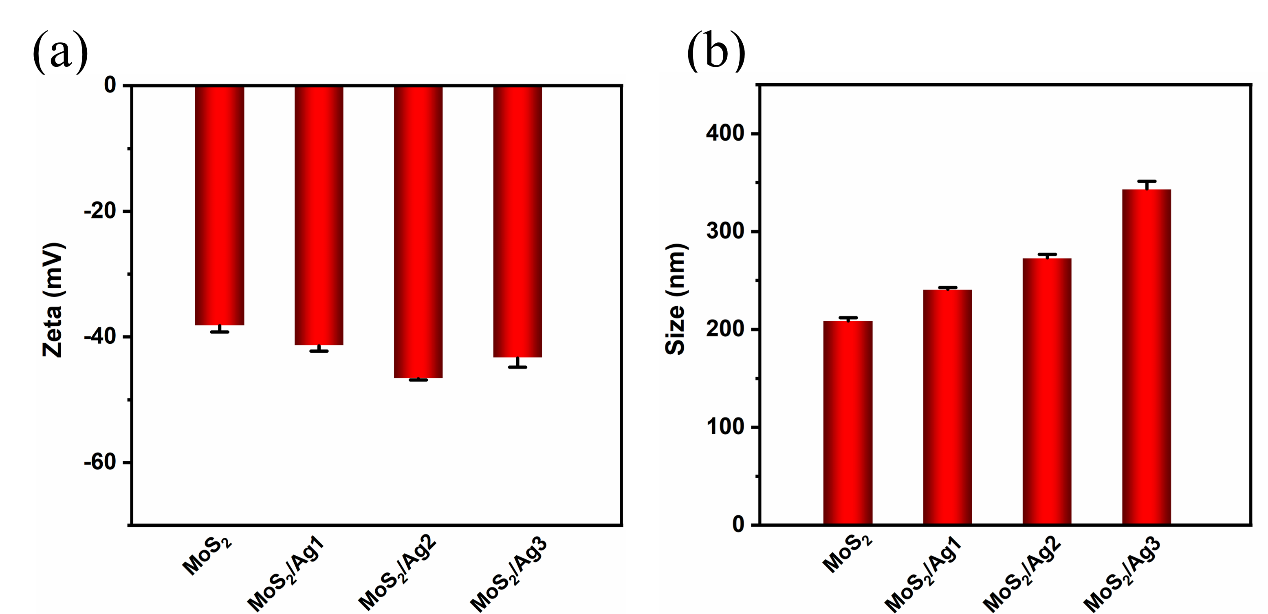


**Fig. S3.** The illustration (a) and (b) of Zeta potential, Size of the different samples (MoS_2_, MoS_2_/Ag1, MoS_2_/Ag2, MoS_2_/Ag3) at the concentration of 25 μg/mL, respectively.





**Fig. S4.** EPR spectra of ^1^O_2_ in aqueous solution pH 7.0 in the presence of H_2_O_2_ (1 M).


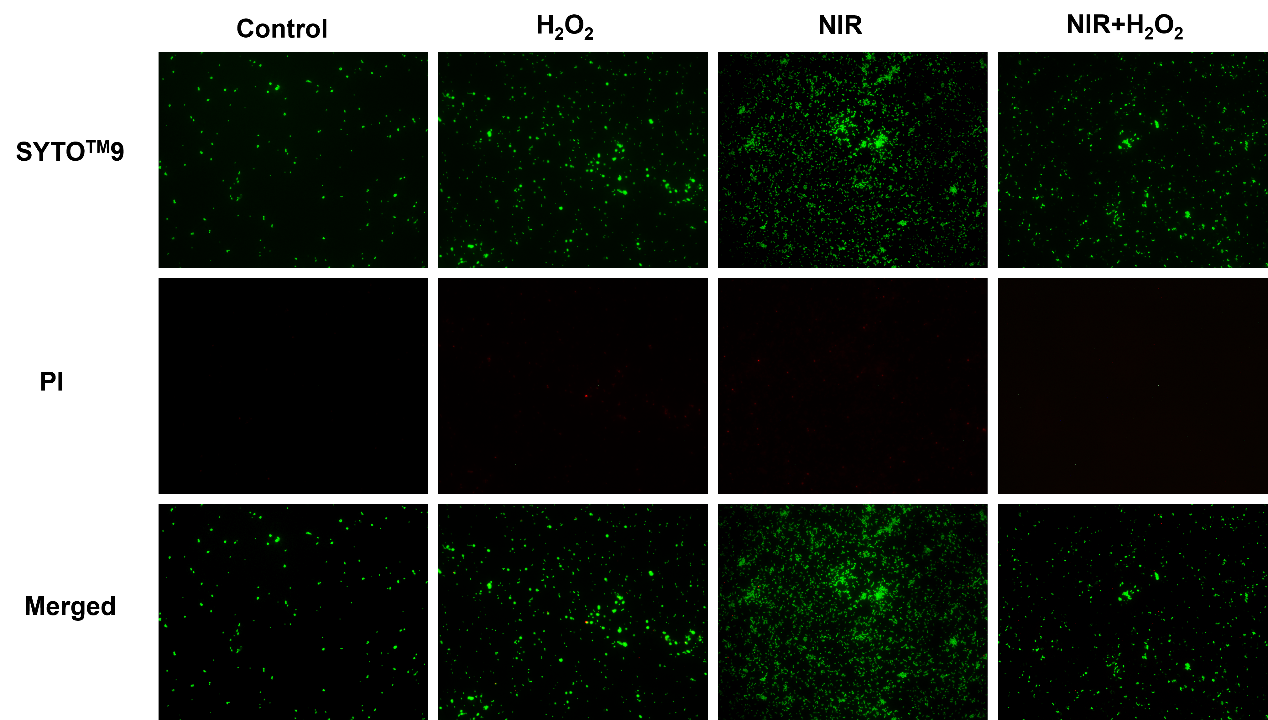


**Fig. S5.** Fluorescent images of *S. aureus* incubated with PBS.
